# Transition to practice: can rural interprofessional education make a difference? A cohort study

**DOI:** 10.1186/s12909-016-0674-5

**Published:** 2016-05-28

**Authors:** Susan (Sue) Pullon, Christine Wilson, Peter Gallagher, Margot Skinner, Eileen McKinlay, Lesley Gray, Patrick McHugh

**Affiliations:** Department Primary Health Care and General Practice, University of Otago, Wellington, P.O.Box 7343, Wellington New Zealand; Medical Education Unit, University of Otago, Wellington, P.O.Box 7343, Wellington New Zealand; School of Physiotherapy, University of Otago, 325 Great King Street, P.O. Box 56, Dunedin, 9054 New Zealand

**Keywords:** Health sciences students, Interprofessional education, Interdisciplinary, Pre-registration, Indigenous health, Rural health

## Abstract

**Background:**

The transition from student to health practitioner at entry-to-practice is complex, requiring critical acquisition of collaborative practice skills. In rural communities where health need is multidimensional, there is potential for multiple intentional collaborative learning objectives to be met concurrently. A five-week, rurally-located, clinically-based interprofessional programme was introduced as a transition-to-practice rotation for final-year, pre-registration health professional students in the professions of dentistry, dietetics, medicine, nursing, pharmacy and physiotherapy. The programme integrated learning objectives in four related domains: interprofessional practice; hauora Māori (Māori health); rural health; long-term condition management. This study investigated student learning experiences over the first two complete years of the programme, comparing responses from participating students with those from a cohort of non-participating peers.

**Methods:**

Using a pre and post quasi-experimental design, respondents from two successive student year cohorts completed questionnaires at the start and end of their final year. Additional survey data were collected from participating students at the end of each rotation.

**Results:**

131 students participated in the programme during 2013–2014. Participating student respondents (55/131;42 %) reported being significantly better prepared than a cohort of 56 non-participating colleagues in many aspects of their understanding of and knowledge about each of four key learning domains. 94 % (123/131) of programme participants completed end-of-rotation questionnaires. Positive from the outset (mean 5-point Likert scale scores between 3 and 5; 5 = most positive), student satisfaction further increased across all domains in the second year (mean 5-point Likert scale scores between 4 and 5).

**Conclusions:**

At entry-to-practice level, multiple learning objectives, including indigenous health learning, can be met simultaneously in the clinical context within an integrated, rotational programme. Rural settings are highly suitable for delivering such programmes if well supported.

## Background

Interprofessional education (IPE) opportunities, particularly if clinically based, aim to enable students to become *collaborative practice-ready* by the time they start work [1]. Collaborative practice-ready graduates are considered to work together more effectively, leading the way towards better, more collaborative and sustainable health systems [2–5].

The transition from senior student to new practitioner is a complex one, typically requiring not only increasing clinical workplace experience and multiple skill acquisition, but equally importantly, critical reflection in context and ongoing dialogue with others [6–8]. The day-to-day reality of busy clinical practice can seem irreconcilable with previous learning unless students are supported in this transition [9]. High quality IPE intentionally facilitates such transformative learning [10–12]; students who have had explicit IPE learning opportunities are increasingly reporting many beneficial flow on effects in multiple learning areas [13, 14]. Effective delivery of clinically-based IPE for pre-registration students has been demonstrated in a variety of settings, with positive outcomes in transitioning to effective collaborative practice [15].

Clinically-based IPE programmes build on well-established principles of community-based education (CBE) [16] (e.g. attending to social and emotional aspects of learning, community values and whole person-orientated care of a generalist nature). The CBE model (often promulgated in rural areas) is recognized as affording students rich opportunities to learn in especially meaningful ways [17], including deepening understanding of health and illness in rural or other contexts, considering the impact of social and environmental factors on health, improving interpersonal skills and gaining understanding of the nature of teamwork [18].

Community-orientated IPE programmes for senior students can go an important step further, intentionally bringing students from different health professions together to not only understand something of interprofessional teamwork [2, 19], but to practise and gain interprofessional competencies around the time they start work as new graduate health professionals.

Interprofessional collaborative practice has particular benefit in rural health care, which can both provide and benefit from IPE. Rural settings show promise as particularly suitable IPE learning environments for those at entry-to-practice level [20]. Paradoxically, IPE is challenging to establish in rural areas, where staff numbers are small and shortages are common, and as yet there are few programmes reporting evaluation results at entry-to-practice level in rural settings. Notable exceptions include some innovative programmes which have reported considerable learning benefits for senior students, strengthening both interprofessional and rural health competencies [10, 21–24].

In rural communities health need is multidimensional (Māori models of health care and often complex healthcare need, highly variable but often low socioeconomic status, difficulties in accessing health services due to availability, distance and poor road access, health literacy, other risk factors for poor health outcomes) [25]. Where students can be immersed in rural clinical practice for a period of time, there is potential for a wide range of intentional learning objectives to be met simultaneously. To make the most of such opportunity without overwhelming students, programmes need to be well-designed and well-facilitated to ensure an appropriate balance between activity and reflection [10].

Integration and evaluation of such clinically-based IPE programmes, within the context of established health professional degree programmes, is challenging. Ideally evaluation is multifaceted, taking into account teacher and student experiences, effect on learning, and effect on whole-of-programme curricula, as well as impact on clinical workplaces and the local community [2, 26].

A clinically-based interprofessional rotational programme for final year pre-registration students was developed for and delivered in a high needs rural area of New Zealand with a high Māori population.

This study investigated and compared learning experiences of participating final year pre-registration students with a cohort of non-participating peers. These findings form a key component of a comprehensive programme evaluation, including community stakeholder, clinical workplace, community, clinical teacher and student feedback [27, 28].

## Methods

### Aim

The study aimed to determine differences between students who participated in a rural interprofessional programme and a cohort of their non-participant peers, and to investigate participant student experiences. Using a pre and post quasi-experimental design, respondents from two successive student year cohorts completed questionnaires at the start and end of their final year. Additional survey data were collected from participating students at the end of each rotation.

### Context

The Tairāwhiti Interprofessional Education (TIPE) programme is based in a remote rural area with a dispersed population of approximately 47,000 [29]. Forty-six percent are Māori, the highest relative number of indigenous peoples per District Health Board (DHB) region in New Zealand [30]. The programme is run by the University of Otago in collaboration with the Eastern Institute of Technology, and the Tairāwhiti and Hawkes Bay DHBs. Specific intended learning outcomes include not only demonstration of well-established interprofessional competencies [31], but also Treaty of Waitangi obligations [32], competency in hauora Māori (Māori health), long-term condition management skills and understanding of the rural health context [33].

Learning is undertaken in five-week clinically-based rotations spaced throughout an academic year. A proportion of students (some self-selected, some allocated, depending on discipline preference and curricula timing options) from each contributing profession (dentistry, dietetics, medicine, nursing, pharmacy and physiotherapy) attend the programme once at some point during their final year of study (TIPE students). Each rotation commences with a formal Māori welcome (including a ‘noho marae’ - overnight stay in a traditional communal living situation). Students live in shared, mixed discipline accommodation, learning with from and about each other and their respective disciplines. A dedicated e-learning platform is available to all students and local staff, incorporating pre-reading, relevant resource material, and facility for online discussion.

Community-based, the integrated programme is explicitly interprofessional, with intended interprofessional learning outcomes agreed across the disciplines for all students. Rural health, long term conditions, hauora Māori and other learning objectives are consistent with and integral to, an overarching IPE framework. Students spend approximately 20 % of their formal learning time undertaking interprofessional classroom or experiential projects, 50–55 % working under supervision in their own clinical disciplines, and 25–30 % of their time working in pairs (occasionally threesomes) in interprofessional clinical placements, in each other’s clinical disciplines. Informal learning continues after hours as they discuss and work further on their interprofessional projects in their shared living situation.

Students work together on case studies, hauora Māori, and community education projects. For the community education projects, students work in interprofessional groups on topics specifically chosen by local community-based healthcare providers. Findings and any relevant resources developed are subsequently formally presented by students to the health care providers.

When working in their own clinical discipline, students greatly value opportunities to take on appropriate clinical responsibility, whereas the scope of their participation (particularly in relation to technical skills) is necessarily more variable when attending clinical work in a discipline other than their own (an interprofessional clinical placement). Regardless, they work with and learn from and about a range of health professionals in their clinical placements – for example, with nurses, doctors and pharmacists in primary care; or with nurses, physiotherapists, dietitians and others in the community-based DHB long term conditions clinic.

The mix of individual and group activities constitutes an integrated interprofessional programme of experiential and reflective learning, as well as any relevant discipline-specific requirements for clinical placements. Programme design, refinements and intended outcomes are agreed by all participating professions via an ongoing, interprofessional, cross-institutional operations group. A high level governance group includes representation from local Māori iwi (local tribal group), education and rural health leaders, and other community stakeholders.

The local teaching team includes a half-time programme leader, a full-time programme administrator, part-time (0.1FTE) clinical educators from each profession, and a hauora Māori teacher. The teaching team work together, having learnt to teach all students using an interprofessional ethos and framework. The programme utilizes as many community and hospital-based health provider services as possible for workplace clinical experience across the region. The local programme leader and administrator oversee the ‘pastoral care’ needs of the students, and manage the clinical placements and other course components.

### Non-participant student context

Students who did not participate in the TIPE programme (non-TIPE students) continued with their usual final year uni-professional course work. All (with the exception of pharmacy students) had uni-professional placements in clinical workplaces of similar or slightly longer length and of a similar community-based nature, often but not always in a regional or rural location. All had hauora Māori teaching components embedded within their respective degree programmes, but few had clinical workplace experience in areas with a comparably high Māori population. At the time of the study there were no alternative IPE programmes comparable with TIPE available to the non-participant student groups.

### Evaluation design

In collaboration with an experienced independent evaluator (Malatest International, New Zealand), a logic model based on Kirkpatrick’s four levels of evaluation for training [34] was developed. The model included evaluation of students’ reactions, resultant learning, behavioural changes and effects on others. A multi-method approach was used to assess the initiation phases of the project from various viewpoints, as well as to investigate impact on the community groups [35]. Teachers’ experiences have been reported elsewhere [28].

Survey data collected from students consisted of a) end-of-rotation questionnaires and b) year-start and year-end questionnaires, not only from students who participated in the TIPE programme (TIPE students), but also from a cohort of comparable peers who had not participated (non-TIPE students). End of rotation focus group data were also collected and analyzed, with results reported previously [27, 36].

The questionnaire design was informed by already-published scales [37] but extended to include measures that were relevant to the programme and New Zealand context. Areas of enquiry included: opportunities to practise clinical skills; understanding of and confidence in aspects of rural health; interprofessional practice; hauora Māori and long-term condition management, and experiences of living and working in a rural community.

### Data analysis

End-of-rotation data were entered into Microsoft Excel for collation and analysis. Questions used a five-point Likert type scale, with free text comments invited. Data were aggregated and 95 % confidence intervals calculated.

Students’ year-start and year-end surveys were matched using student identification numbers (either using their National Student Number or Tertiary Institute Identification number), student names, email addresses and phone numbers. The dataset of the survey responses from students who completed both surveys served as the primary data source for the comparison of learning outcomes between TIPE and non-TIPE students.

Three analyses were conducted for each question using SPSS V20.0 for Windows software (SPSS, Inc, Chicago, Illinois, USA). Descriptive statistics identified the year-start and year-end means for both groups of students. Means were compared using paired samples T-tests for students who answered both surveys. Repeated measures analysis of variance (ANOVA) was used to identify whether the two groups of students changed differently over time.

Results are presented for the analysis of variance based on matched year-start and year-end survey responses. The results from comparison between all year-start and year-end responses (unmatched) were consistent, but the paired results comparison was more powerful despite the smaller sample size.

Free text data were collated and reviewed. Data were then examined for similarity, differences and complementarity.

## Results

### Participation

#### Professions

One hundred and thirty one students from seven health professions participated in the TIPE programme over the study period (2013–2014). Rotations averaged 12–13 students each (Table 1). Results reported are from student questionnaire data collection undertaken over the first two complete years of the programme (10 consecutive rotations).

**Table 1:** Numbers of students participating in the TIPE programme by health profession 2013-2014

Rotational blocks	Dentistry	Dietetics	Medicine	Nursing	OT	Pharmacy	Physiotherapy	All students
2013; *n* = 60								
1	2	2	3	0		3	2	12
2	0	2	2	3		3	2	12
3	2	2	2	4		0	2	12
4	2	2	3	0		3	2	12
5	2	2	3	3		0	2	12
2014; *n* = 71								
1	2	1	1	0		4	2	10
2	2	2	3	4		3	2	16
3	2	2	3	5		0	2	14
4	2	2	3	0	2	4	0	13
5	2	2	4	6	2	0	2	18
Totals	18	19	27	25	4	20	18	131

Occupational therapy (OT) students 2014 not included in comparative analyses (Tables 4 and 5)

### Student evaluation - year start and year end results

Year-start and year-end questionnaires were conducted over the study period, comparing TIPE students with a cohort of their non-TIPE peers. A total of 111 students answered both the year-start and year-end surveys across 2013–2014. A larger number of students completed only the year-start (326/1183;28 %) or only the year-end questionnaires (255/1183;22 %), but these responses were excluded from further analysis. Year-start and year-end responses from 55 TIPE students (55/131;42 %) were able to be compared with those from 56 non-TIPE students in the same year cohorts.

Students participating in the TIPE programme reported significant increases in their understanding and knowledge about key aspects of interprofessional working and practice, rural health, Māori culture and customs, and long-term condition management between the start and end of each year. The changes were also significantly greater than those reported from non-TIPE students over the same period (Table 2).

**Table 2:** Change in students’ agreement between the year-start and year-end surveys 2013–2014, TIPE (*n* = 55) and non-TIPE students (*n* = 56)

Student agreement with the following statements	Year-start TIPE	Year-end TIPE	YS-YE TIPE difference	Year-start non-TIPE	Year-end non-TIPE	YS-YE non-TIPE difference	Difference in TIPE/non-TIPE change
	TIPE			Non-TIPE			
Interprofessionality (IP) – to what extent do you agree with the statements?							
I understand the roles, activities and skills of different healthcare professionals	3.09	4.55	+1.45	3.25	3.5	+.25	1.20*
I am comfortable working with people from other health care disciplines	4.02	4.73	+.71	4.07	4.09	+.02	0.69*
An IP approach permits health professional to meet the needs of patients	4.58	4.89	+.31	4.63	4.36	-.27	0.58*
An IP approach better meets the needs of family, caregivers and parents	4.58	4.91	+.33	4.54	4.38	-.16	0.49*
An IP approach improves the quality of care to patients/clients	4.73	4.95	+.22	4.64	4.41	-.23	0.45*
Rural health care – to what extent do you…							
understand the roles of IP teams in rural health care	2.07	4.25	+2.18	2.46	2.73	+.27	1.91*
understand barriers to care in rural areas	2.78	4.49	+1.71	3.05	3.25	+.2	1.51*
understand what works well in rural health care	1.89	3.91	+.2.02	2.13	2.5	+.38	1.64*
understand the community roles of rural health care workers	2.25	4.2	+1.95	2.46	2.73	+.27	1.68*
Hauora Maori – how do you rate…							
…your knowledge about Maori culture and customs	2.76	4.09	+1.33	2.68	3.00	+.32	1.01*
The impact of whanau ora on health care provision	2.8	4.36	+1.56	2.75	3.02	+.27	1.29*
The impact of family dynamics on health care decisions	3.04	4.49	+1.45	3.21	3.39	+.18	1.27*
The impact of the social environment on community health	3.42	4.38	+.96	3.64	3.57	-.07	1.03*
High needs chronic condition management – what is your understanding about…							
Education resources for patients with chronic conditions	2.71	3.96	+1.25	2.86	3.21	+.36	0.89*
Health promotion approaches to prevent chronic conditions	3.07	4.05	+.98	3.30	3.32	+.02	0.96*
Evidence based guidelines to managing chronic conditions	2.87	3.80	+.93	2.93	3.09	+.16	0.77*

TIPE changes are marked with an asterisk when they are significantly different (*p* < 0.05) from non-TIPE students. (OT students not included)

TIPE students’ agreement with statements about the roles, activities and skills of other professionals increased significantly more than non-TIPE students in both their comfort working with (F(1,109) = 16.740, *p* < 0.001) and their understanding of other professionals (F(1,109) = 35.351, *p* < 0.001). Furthermore, TIPE students increased their agreement significantly more than their non-TIPE peers that an interprofessional approach improves quality of care to patients/clients (F(1,109) = 12.950, *p* < 0.001); better meets the needs of family, caregivers and patients (F(1,109) = 12.490, *p* < 0.01), and permits health professionals to meet the needs of patients (F(1,109) = 15.554, *p* < 0.001).

The TIPE students also reported significantly more improvement in their knowledge about aspects of rural healthcare than their non-participating peers with respect to the community roles of rural healthcare workers (F(1,109) = 62.672, *p* < 0.001); what works well in rural healthcare (F(1,109) = 64.757, *p* < 0.001); barriers to health care in rural areas (F(1,109) = 58.557, *p* < 0.001) and the roles of interprofessional teams in rural healthcare (F(1,109) = 79.615, *p* < 0.001). The TIPE students increased their knowledge significantly more than the non-TIPE students regarding the impact of social and environmental conditions on the health and wellbeing of communities (F(1,51) = 21.087, *p* < 0.001); the impact of family dynamics on healthcare decisions (F(1,51) = 30.358, *p* < 0.001); the impact of whānau ora (Māori model of family-centred care) on healthcare provision (F(1,51) = 19.148, *p* < 0.001) and Māori culture and customs (F(1,51) = 26.277, *p* < 0.001).

### Student evaluation – end of rotation results

Of the TIPE students participating in the programme in the study period, ninety four percent (123/131) responded, completing questionnaires at the end of their rotation. Overall satisfaction with the programme was high, (mean 5-point Likert scale scores between 3 and 5; 5 = most positive), with student satisfaction further increasing across programme objectives and learning domains in the second year (mean 5-point Likert scale scores between 4 and 5) - (Table 3). Opportunities for effective interprofessional learning in the rural setting were well regarded from the outset, with TIPE students strongly agreeing that their knowledge of and confidence in working interprofessionally across the health disciplines, and their understanding of the rural health environment, improved a great deal over the course of the 5-week rotation. Students also agreed that their knowledge of Māori culture and customs, and ability to incorporate these values and skills into their practice had greatly improved as a result of the programme. In contrast, opportunities for skill acquisition in aspects of long-term condition management were slower to build up as the programme developed, with mixed results from the 2013 cohort, but by 2014, students reported high satisfaction with this aspect of the programme.

**Table 3:** Aggregated responses from 123/131 TIPE students who completed end-of-rotation questionnaires 2013–2014. Responses on a 5-point Likert-type scale where 5 = strongly agree/excellent;1 = strongly disagree/poor

Questions	**Mean response for item**	
	95 % confidence intervals	
	2013	2014
	*N* = 53	*N* = 70
**Satisfaction with the programme**		
I enjoyed taking part in the TIPE programme	**4.5**	**4.8**
	4.3–4.7	4.6–4.9
I would recommend the programme to other students	**4.2**	**4.7**
	3.8–4.5	4.6–4.9
I felt included as part of the Tairāwhiti community	**4.6**	**4.6**
	4.4–4.8	4.5–4.8
**Hauora Maori**		
I have a better understanding about how I can contribute to improve Māori health.	**4.3**	**4.6**
	4.0–4.5	4.4–4.7
I have a better understanding of how to incorporate Māori culture and customs into my practice.	**4.3**	**4.6**
	4.0–4.5	4.5–4.8
I feel more confident that I can provide care to Māori patients.	**4.3**	**4.6**
	4.0–4.5	4.5–4.8
**Rural health**		
I have become more confident in practising in rural health.	**4.3**	**4.6**
	4.1–4.5	4.5–4.7
I have become more knowledgeable about the roles of other health professionals	**4.3**	**4.7**
	4.1–4.5	4.6–4.8
**Interprofessional/interdisciplinary health care**		
My skills in communicating with other health and social care professionals improved through learning with students from other healthcare professions.	**4.3**	**4.6**
	4.1–4.6	4.4–4.7
Learning with students from other healthcare professions is more beneficial to improving teamwork skills than learning only with peers.	**4.5**	**4.7**
	4.2–4.7	4.6–4.9
As a result of the programme I have a better understanding of the roles, activities and skill of different healthcare professionals.	**4.5**	**4.7**
	4.3–4.7	4.6–4.9
**Chronic condition management**		
My knowledge about organizing care for people with chronic conditions	**3.6**	**3.9**
	3.3–3.9	3.7–4.2
My knowledge of what other professions can contribute to chronic conditions care	**3.9**	**4.4**
	3.6–4.2	4.2–4.6
My ability to work collaboratively with other disciplines to organize care for people with chronic conditions	**3.8**	**4.3**
	3.5–4.1	4.1–4.5
**Learning on clinical placements**		
I had sufficient opportunity to practise my clinical skills.	**3.5**	**3.8**
	3.2–3.9	3.5–4.0
The clinical teachers were sensitive and responsive to patients and their relatives.	**4.5**	**4.6**
	4.3–4.7	4.4–4.8
The clinical teachers were sensitive and responsive to other health professionals.	**4.4**	**4.6**
	4.1–4.6	4.4–4.8
I was encouraged to think through clinical problems for myself.	**4.2**	**4.6**
	4.0–4.4	4.4–4.7
I was given adequate instructions for proceeding with clinical work.	**4.2**	**4.3**
	3.9–4.4	4.1–4.5

(2013 *n* = 53/60; 2014 *n* = 70/71; total *n* = 123/131) 94 % response rate. Mean values bold for emphasis

Free-text comments reiterated the positive learning experiences the programme as a whole afforded. The integration of various objectives into the one programme was often mentioned; with the interprofessional, shared nature of the learning being a significant point of difference to other parts of their various degree courses. Table 4 gives a sample of student comments.

**Table 4:** Examples of TIPE student responses to end-of-rotation free text questions, 2013-2014

What did you like most about the programme?
*Loved placements in other professions and getting to know everyone outside the classroom (Student, block 2, 2013)*
*Opportunities we would never have at school, gaining skills in communicating with other professionals. I could overcome stereotype towards rural area and Maori culture and health (Student, block 4, 2013)*
*Working in a rural community where everyone is friendly, living with other IPE students, gaining greater knowledge of other professions, so much respect for other health professionals (Student, block 5, 2013)*
*Living with all the professions, the 2 nursing placements really interesting, seeing first hand so many clinical situations made my degree relevant (Student, block 1, 2014)*
*Living with a mixture of health students and get to know more about what they do and understand their perceptions on healthcare. Breaking the stereotypical view on what they do personally the clinical placements of other professions (Student, block 1, 2014)*
*The cultural immersion up here was a great experience (Student, block 2, 2014)*
*Being able to understand more about other professions which broke down barriers. Also learnt about dynamics of rural health (student, block 2, 2014)*
*…learning to work in a rural setting, interacting with a small community, working with Maori patients*
*living with other IPE students, Clinical placement excellent, Hauora [Maori] aspects good (Student block 3,2014)*
*Being part of a community largely Maori and living with observing what other health professionals do*
*Working with other health professionals, learning about other disciplines and how pharmacy can be better with them. Challenging clinical placements, Group projects (Student, block 4, 2014)*
What improvements would you like to see?
*Not enough clinical time in my own profession, quite a bit of wasted time ie IPE placements that were unorganised/unsure of purpose. Done a lot of nursing placements already (Student, block 4, 2013)*
*Would put clinical placements side by side not disjointed 1/2 days, Call placements to remind them that we are coming (Student, block 5, 2013)*
*The quality of accommodation could be improved, lack of kitchen equipment, hospital pillows, and no coat hangers (Student, block 2, 2014)*
*…slightly more clinical time, some of the teaching sessions could be better organised, Vivid [videoconference] hard (Student, block 3, 2014)*
*[not]Getting a chance to visit all the [other] professions (Student, block 4, 2014)*

Some professional differences were evident. There were some apparent differences in relation to some aspects of the programme, particularly clinical work. To some extent, dental and medical students reported less satisfaction than students from other professions with their opportunities for practising discipline-specific skills in 2013, although there was little difference in mean scores across the professions by 2014 (Table 5). Given some dental and medical student anxiety about opportunities for adequate technical clinical skill acquisition in a transition-to-practice phase of training [38], key programme objectives were clarified in 2014 for all TIPE students to further emphasise the multifaceted nature of patient-centred care, and the value of cultural, interprofessional and rural competencies alongside core clinical competencies.

**Table 5:** Responses by professional group from end-of-rotation questionnaires completed by 119/127 students who participated in the TIPE programme 2013–2014 (4 OT students not included). Responses on a 5-point Likert-type scale where 1 = strongly disagree/poor; 5 = strongly agree/excellent

Student agreement with the following statements	Dentistry *N* = 18	Dietetics *N* = 17	Medicine *N* = 23	Nursing *N* = 25	Pharmacy *N* = 18	Physiotherapy *N* = 18
	**Mean**	**Mean**	**Mean**	**Mean**	**Mean**	**Mean**
	Confidence interval 95 %	Confidence interval 95 %	Confidence interval 95 %	Confidence interval 95 %	Confidence interval 95 %	Confidence interval 95 %
**Satisfaction with the Programme**						
I enjoyed taking part in the TIPE programme	**4.4**	**4.9**	**4.1**	**4.8**	**4.8**	**4.9**
	4.0 -4.8	4.8–5.1	3.6 -4.6	4.6–4.9	4.6–5.0	4.8–5.1
I would recommend the programme to other students	**3.9**	**4.9**	**3.7**	**4.7**	**4.8**	**4.9**
	3.4–4.4	4.7–5.1	3.0–4.3	4.5–5.0	4.6–5.0	4.8–5.1
I felt included as part of the Tairawhiti community	**4.4**	**4.9**	**4.7**	**4.4**	**4.6**	**4.8**
	4.1- 4.8	4.7–5.1	4.5–4.9	4.1–4.8	4.2–5.0	4.6–5.0
**Hauora Maori**						
I have a better understanding about how I can contribute to improve Māori health.	**4.3**	**4.6**	**3.8**	**4.6**	**4.5**	**4.7**
	4.0–4.7	4.4–4.9	3.4–4.3	4.4–4.9	4.2–4.7	4.4–4.9
I have a better understanding of how to incorporate Māori culture and customs into my practice.	**4.3**	**4.8**	**3.9**	**4.8**	**4.6**	**4.6**
	4.0–4.6	4.5–5.0	3.5–4.3	4.6–5.0	4.3–4.8	4.2–4.9
I feel more confident that I can provide care to Māori patients.	**4.3**	**4.6**	**4.0**	**4.7**	**4.4**	**4.7**
	4.0–4.6	4.2–5.1	3.6–4.4	4.5–5.0	4.1–4.7	4.5–5.0
**Rural Health**						
I have become more confident in practising in rural health.	**4.1**	**4.4**	**4.3**	**4.6**	**4.5**	**4.8**
	3.7–4.6	4.0–4.8	4.0–4.6	4.3–4.9	4.2–4.8	4.6–5.0
I have become more knowledgeable about the roles of other health professionals	**4.5**	**4.8**	**4.2**	**4.7**	**4.6**	**4.8**
	4.2–4.8	4.6–5.0	3.9–4.6	4.5–5.0	4.3–4.9	4.6–5.0
**Interprofessional Health Care**						
My skills in communicating with other health and social care professionals improved through learning with students from other healthcare professions.	**4.4**	**4.6**	**3.9**	**4.6**	**4.6**	**4.6**
	4.1–4.7	4.3–5.0	3.5–4.3	4.4–4.9	4.4–4.8	4.4–4.9
Learning with students from other healthcare professions is more beneficial to improving teamwork skills than learning only with peers.	**4.6**	**4.8**	**4.1**	**4.7**	**4.7**	**4.9**
	4.2–4.9	4.6–5.0	3.7–4.6	4.4–5.0	4.4–5.0	4.7–5.0
As a result of the programme I have a better understanding of the roles, activities and skill of different healthcare professionals.	**4.5**	**4.8**	**4.3**	**4.8**	**4.8**	**4.8**
	4.2–4.8	4.6–5.0	3.8–4.6	4.5–5.0	4.6–5.0	4.6–5.0
**Chronic Condition Management**						
My knowledge about organizing care for people with chronic conditions	**3.4**	**3.9**	**2.8**	**4.3**	**4.2**	**4.0**
	2.8–4.0	3.4–4.4	2.3–3.3	3.9–4.7	3.98–4.6	3.7–4.3
My knowledge of what other professions can contribute to chronic conditions care	**4.0**	**4.3**	**3.3**	**4.6**	**4.5**	**4.3**
	3.4–4.6	3.8–4.8	2.8–3.8	4.3–4.9	4.1–4.9	3.8–4.8
My ability to work collaboratively with other disciplines to organize care for people with chronic conditions	**3.7**	**4.0**	**3.3**	**4.6**	**4.5**	**4.3**
	3.2–4.3	3.6–4.4	2.8–3.8	4.4–4.9	4.2–4.8	3.9–4.8
**Learning on Clinical Placements**.						
I had sufficient opportunity to practise my clinical skills.	**2.9**	**4.2**	**3.7**	**3.4**	**3.8**	**4.2**
	2.2–3.6	3.8–4.6	3.2–4.2	2.9–3.9	3.4–4.3	3.9–4.4
The clinical teachers were sensitive and responsive to patients and their relatives.	**4.1**	**4.6**	**4.6**	**4.6**	**4.4**	**4.8**
	3.6–4.6	4.3–4.9	4.3–4.8	4.3–4.9	3.9–4.8	4.6–5.0
The clinical teachers were sensitive and responsive to other health professionals.	**3.9**	**4.5**	**4.4**	**4.6**	**4.6**	**4.7**
	3.4–4.4	4.3–5.0	4.1–4.7	4.3–4.9	4.3–4.9	4.5–5.0
I was encouraged to think through clinical problems for myself.	**4.3**	**4.6**	**4.6**	**4.1**	**4.2**	**4.7**
	3.9–4.7	4.4–4.9	4.3–4.8	3.7–4.5	3.7–4.6	4.4–4.9
I was given adequate instructions for proceeding with clinical work.	**3.8**	**4.2**	**4.2**	**4.2**	**4.2**	**4.7**
	3.4–4.3	3.8–4.6	3.7–4.7	3.9–4.6	3.8–4.6	4.4–5.0

(119/127 students; 94 % response rate). Mean values bold for emphasis

## Discussion

Students participating in a final year interprofessional transition-to-practice programme reported being significantly better prepared than their non-participant colleagues in understanding of and knowledge about collaborative practice, rural health care, hauora Māori and the management of those with long-term conditions. While some non-participant students also had clinically-based rural experience, experience working with Māori and exposure to long term condition management, this was largely opportunistic, and was almost exclusively uni-professional. The explicit interprofessional learning objectives and outcomes of TIPE were thus a key point of difference from other clinically-based learning.

Dental and medical students initially appeared to consider that their discipline-specific learning (particularly technical skills) might be compromised by the need to meet other objectives, although these concerns decreased in successive rotations as each academic year progressed. Remarkably few other studies have shown that that multiple learning objectives, including indigenous health learning, can be met simultaneously during an interprofessional, rotational programme delivered in a rural area. In response to student feedback, progressive refining of programme delivery occurred over two years, resulting in increased student satisfaction over successive groups.

Although there was a good response rate from TIPE students to successive end-of-rotation questionnaires, the portion of the study comparing TIPE students with their non-TIPE peers was limited by the low response rate from non-TIPE students in the year-start and year-end surveys, and by the number of students who did not complete both the year-start and year-end surveys. Despite this important limitation, analysis highlighted statistically some significant differences between TIPE students and non-TIPE peers. It was not possible to gather information about any differences between students who answered both surveys and those who answered neither survey. However, comparison between the students who answered both surveys and those who only answered one (either the pre- or post-survey) showed the samples were broadly consistent. Analyses of successive end of rotation focus group data from the TIPE students (with similar response rates to the end of rotation questionnaires) have been previously reported [27, 36], further supporting and extending the findings of this study.

There are few other reported studies of transition-to-practice rurally located, clinically-based interprofessional programmes of several weeks duration. Our study findings regarding student satisfaction are most comparable with results from rural, entry-to-practice level interprofessional programmes in Ontario, and British Columbia, Canada [10, 21], and in Illinois, USA [39]. Results from pre- and post-programme student questionnaires for each indicated improved interaction with and understanding of students from other professions over the course of the programmes. Although shorter in duration, the 2-week Rural Interprofessional Education project in Victoria, Australia [40] was also comparable, showing not only positive changes over the course of the programme but also professional differences [23]. While these programmes had explicit interprofessional and rural health learning objectives, none described specific and additional learning objectives about indigenous health or long-term condition management. In contrast, the Community Week programme in Western Australia [12], and a more recently reported South African programme [24], have also identified the importance of engaging with indigenous communities when delivering rurally based interprofessional education.

Participant pre and post questionnaires have been employed in studies of other rurally-based interprofessional learning activities, and have demonstrated positive change, but comparison with our study is more limited as these were either classroom-based, and/or not at entry-to-practice level [22, 41]. No other comparable rurally-based programme has reported comparative data from a non-participating peer cohort; in this respect our study findings are unique.

Although not rurally based, the Linköping model of IPE, where senior students learn together caring for patients on an interprofessional ward, is an entry-to-practice level programme. Medical students from this programme were compared to their non-participant peers post-graduation, and reported significantly more confidence in working with others in health care teams, a trait that persisted over several years [38]. This study also found no impact, adverse or otherwise, from IPE participation on acute care medical skills in new medical graduates, an important consideration in the light of early concerns about reduced opportunities for clinical practice raised initially by dental and medical students in our study.

## Conclusion

The results from this innovative New Zealand study extend knowledge in several respects about transition-to-practice education internationally, particularly for countries with comparable health education systems. Rurally-based IPE at the entry-to-practice phase of training can meet multiple objectives in preparing participating students for practice, over and above otherwise equivalent education for their non-participating peers. Clinically-based interprofessional programmes are challenging to set up in small communities with small health workforces, but as local clinical teachers are informed by student feedback, and become interprofessionally skilled, delivery becomes easier over time, enhancing student learning and satisfaction. If multiple learning objectives can be met concurrently in well supported rural IPE programmes, learning outcomes can be maximised for a wide range of health professional students in ways that are sustainable and beneficial for local communities.

## Abbreviations

CBE, community –based education; DHB, District Health Board; hauora Māori, Māori health; IPE, InterProfessional Education; Non-TIPE, students who did not participate in the TIPE programme; TIPE, Tairāwhiti InterProfessional Education (programme).
